# Utilization of Host-Derived Glycans by Intestinal *Lactobacillus* and *Bifidobacterium* Species

**DOI:** 10.3389/fmicb.2018.01917

**Published:** 2018-08-17

**Authors:** Manuel Zúñiga, Vicente Monedero, María J. Yebra

**Affiliations:** Laboratorio de Bacterias Lácticas y Probióticos, Departamento de Biotecnología de Alimentos, Instituto de Agroquímica y Tecnología de Alimentos-Consejo Superior de Investigaciones Científicas, Valencia, Spain

**Keywords:** human milk oligosaccharides (HMOs), mucins, glycolipids, glycosaminoglycans, *Lactobacillus*, *Bifidobacterium*, glycosidases

## Abstract

Members of the genus *Lactobacillus* are commonly found at the gastrointestinal tract and other mucosal surfaces of humans. This genus includes various species with a great number of potentially probiotic bacteria. Other often-used probiotic species belong to *Bifidobacterium*, a genus almost exclusively associated with the gut. As probiotics must survive and be metabolically active at their target sites, namely host mucosal surfaces, consumption of host-produced glycans is a key factor for their survival and activity. The ability to metabolize glycans such as human milk oligosaccharides (HMOs), glycosaminoglycans and the glycan moieties of glycoproteins and glycolipids found at the mucosal surfaces grants a competitive advantage to lactobacilli and bifidobacteria. The analyses of the great number of sequenced genomes from these bacteria have revealed that many of them encode a wide assortment of genes involved in the metabolism and transport of carbohydrates, including several glycoside hydrolases required for metabolizing the carbohydrate moieties of mucins and HMOs. Here, the current knowledge on the genetic mechanisms, known catabolic pathways and biochemical properties of enzymes involved in the utilization of host-produced glycans by lactobacilli and bifidobacteria will be summarized.

## Introduction

Microbes colonize every available body surface area where they establish symbiotic relationships among themselves and with the host. Microbes affect nearly all aspects of human physiology to such extent that their absence may result in severe nutritional, developmental or defensive dysfunctions. Gastrointestinal (GI) microbiota constitute the most abundant microbial community associated to humans and, consequently, has engaged the attention of the scientific community. As a result of this effort, solid evidence has been obtained demonstrating that gut microbiota exerts major effects on human physiology, from metabolism (Li et al., 2008) to behavior (Ezenwa et al., 2012). Furthermore, alterations of this microbial community may have significant effects on the host health (Cho and Blaser, 2012; Claesson et al., 2012) and have been related to a number of diseases such as metabolic disorders (Sonnenburg and Backhed, 2016), inflammatory diseases (Blander et al., 2017), diabetes (Membrez et al., 2008; Vaarala et al., 2008) and coeliac disease (Collado et al., 2007), among others.

The gut microbiota is a dynamic community whose composition can be altered both by external factors such as food intake (Kohl et al., 2014) and composition (David et al., 2014), and by internal factors (Lozupone et al., 2012) such as the host repertoire of mucins, antimicrobial peptides and immunoglobulins (Rawls et al., 2006; Staubach et al., 2012; Arike and Hansson, 2016). Gut microbes obtain their energy from diet components and host-derived compounds, mainly glycans. The intestinal mucus is a source of carbohydrates readily used by the gut microbiota (Tailford et al., 2015). Reliance on host-derived glycans for growth is possibly more important for other microbial communities associated to humans. For example, proliferation of lactobacilli in the human vagina depends on host-secreted glycogen and α-amylase (Spear et al., 2014). Milk-associated microbiota readily utilize lactose and milk oligosaccharides for growth (Bode, 2012; Jost et al., 2015).

Glycans constitute a large and diverse group of molecules, as the numerous bonding sites of their constituent monosaccharides allow their assembly among themselves or to almost any other organic molecule in an extensive array of architectures (**Tables 1, 2**). Because of this structural versatility, carbohydrates fulfill a wide variety of functions in organisms as structural polymers, energy reserve, signaling, etc. The ability of different bacterial strains to utilize these carbon sources is usually limited to a subset of them. Host organisms may utilize this characteristic to exert some control on the composition of their associated microbiota. Researchers were aware of this fact since the 1950s when the “bifidus factor,” a component of human milk that boosted the growth of bifidobacteria, was described (György et al., 1954; Hutkins et al., 2016). Later on, it provided the basis for the concept of prebiotic, defined as a non-digestible food ingredient that beneficially affects the host by selectively stimulating the growth and/or activity of one or a limited number of bacteria in the colon, and thus improves host health (Gibson and Roberfroid, 1995). Although the definition has been revised and the concept of prebiotic as a selective nutrient is currently discussed (Hutkins et al., 2016), the original idea gave a strong boost to the study of the glycan catabolic pathways of bifidobacteria and lactobacilli.

**Table 1:** Human milk oligosaccharides described in this review.

Name	Abbreviation	Structure
Lacto-*N*-biose	LNB	Galβ1-3GlcNAc
*N*-acetyllactosamine	LacNAc	Galβ1-4GlcNAc
Lacto-*N*-triose II		GlcNAcβ1-3Galβ1-4Glc
Lacto-*N*-tetraose	LNT	Galβ1-3GlcNAcβ1-3Galβ1-4Glc
Lacto-*N*-neotetraose	LNnT	Galβ1-4GlcNAcβ1-3Galβ1-4Glc
Lacto-*N*-hexaose	LNH	Galβ1-3GlcNAcβ1-3(Galβ1-4GlcNAc β1-6)Gal β1-4Glc
3′-Sialyllactose	3SL	Neu5Acα2-3Galβ1-4Glc
6′-Sialyllactose	6SL	Neu5Acα2-6Galβ1-4Glc
Sialyllacto-*N*-tetraose a	LSTa	Neu5Acα2-3Galβ1-3GlcNAcβ1-3-Galβ1-4Gal
Disialyl-lacto-*N*-tetraose	DSLNT	Neu5Acα2-3Galβ1-3(Neu5Acα2-6)GlcNAcβ1-3Galβ1-4Glc
Di-fucosylated sialylated- lacto-*N*-hexaose	DFS-LNH	Fucα1-2Galβ1-3(Fucα1-4)GlcNAcβ1-3(Neu5Acα2-6Galβ1-4GlcNAcβ1-6)Galβ1-4Glc
Fucosylα1-3GlcNAc	3FN	Fucα1-3GlcNAc
Fucosylα1-6GlcNAc	6FN	Fucα1-6GlcNAc
2′-Fucosyllactose	2FL	Fucα1-2Galβ1-4Glc
3′-Fucosyllactose	3FL	Galβ1-4(Fucα1-3)Glc
Lacto-*N*-fucopentaose I	LNFPI	Fucα1-2Galβ1-3GlcNAcβ1-3Galβ1-4Glc
Lacto-*N*-fucopentaose II	LNFPII	Galβ1-3(Fucα1-4)GlcNacβ1-3Galβ1-4Glc
Lacto-*N*-fucopentaose III	LNFPIII	Galβ1-4(Fucα1-3)GlcNacβ1-3Galβ1-4Glc


*Gal, galactose; GlcNAc, N-acetylglucosamine; Glc, glucose; GalNAc, N-acetylgalactosamine; Neu5Ac, N-acetylneuraminic acid; Fuc, fucose.*

**Table 2:** Conjugated glycans described in this review.

Name	Abbreviation	Structure
***O*-Glycans**		
Galacto-*N*-biose	GNB	Galβ1-3GalNAc
Core type 1		Galβ1-3GalNAcα1Ser/Thr
Core type 2		Galβ1-3(GlcNAcβ1-6)GalNAcα1-Ser/Thr
Core type 3		GlcNAcβ1-3GalNAcα1Ser/Thr
Core type 4		GlcNAcβ1-6(GlcNAcβ1-3)GalNAcα1Ser/Thr
***N*-Glycans**		
Core		Manα1-3(Manα1-6)Manβ1-4GlcNAcβ1-4GlcNAcβ1-Asn
*N*,*N*′-diacetylchitobiose	ChbNAc	GlcNAcβ1-4GlcNAc
**Glycosphingolipids**		
Disialoganglioside	GD3	Neu5Acα2-8Neu5Acα2-3Galβ1-4Glcβ1-1Cer
Monosialoganglioside	GM3	Neu5Acα2-3Galβ1-4Glcβ1-1Cer
Monosialoganglioside	GM2	GalNAcβ1-4(Neu5Acα2-3)Galβ1-4Glcβ1-1Cer
Monosialoganglioside	GM1	Galβ1-3GalNAcβ1-4(Neu5Acα2-3)Galβ1-4Glcβ1-1Cer
Fucosyl-GM1		Fucα1-2Galβ1-3GalNAcβ1-4(Neu5Acα2-3)Galβ1-4Glcβ1-1Cer
Globotriaosylceramide	Gb3	Galα1-4Galβ1-4Glβ1-1Cer
Globotetraosylceramide	Gb4	GalNAcβ1-3Galα1-4Galβ1-4Glcβ1-1Cer
Gb5 pentasaccharide	Gb5	Galβ1-3GalNAcβ1-3Galα1-4Galβ1-4Glc
Globo H hexasaccharide	Globo H	Fucα1-2Galβ1-3GalNAcβ1-3Galα1-4Galβ1-4Glc
GA1 tetrasaccharide	GA1	Galβ1-3GalNAcβ1-4Galβ1-4Glc
**Glycosaminoglycans^∗^**		
Hyaluronic acid	HA	GlcAβ1-3GlcNAcβ1-4(GlcAβ1-3GlcNAcβ1-4)_n_GlcAβ1-3GlcNAc
Chondroitin sulfate	CS	GlcAβ1-3GalNAcβ1-4(GlcAβ1-3GalNAcβ1-4)_n_GlcAβ1-3GalNAc
Dermatan sulfate	DS	IdoAα1-3GalNAcβ1-4(IdoAα1-3GalNAcβ1-4)_n_IdoAα1-3GalNAc
Heparin	Hep	GlcAα1-4GlcNα1-4(GlcAα1-4GlcNα1-4)_n_GlcAα1-4GlcN; IdoAβ1-4GlcNα1-4(IdoAβ1-4GlcNα1-4)_n_IdoAβ1-4GlcN
Heparan sulfate	HS	GlcAβ1-4GlcNAcα1-4(GlcAβ1-4GlcNAcα1-4)_n_ GlcAβ1-4GlcNAc; GlcAβ1-4GlcNα1-4(GlcAβ1-4GlcNα1-4)_n_GlcAβ1-4GlcN; IdoAα1-4GlcNα1-4(IdoAα1-4GlcNα1-4)_n_IdoAα1-4GlcN
Keratan sulfate	KS	Galβ1-4GlcNAcβ1-3(Galβ1-4GlcNAcβ1-3)_n_Galβ1-4GlcNAc


*Gal, galactose; GlcNAc, N-acetylglucosamine; Glc, glucose; GalNAc, N-acetylgalactosamine; Neu5Ac, N-acetylneuraminic acid; Fuc, fucose; Cer, ceramide; GlcA, D-glucoronic acid; IdoA, L-iduronic acid; GlcN, D-glucosamine. ***^∗^***The showed structure of glycosaminoglycans backbone can be sulfated.*

*Lactobacillus* are Gram-positive, microaerophilic or anaerobic obligate fermentative organisms that produce lactic acid as the major end product of sugar fermentation. The genus currently comprises over 200 species that have been isolated from a wide variety of habitats. Together with genera *Paralactobacillus*, *Pediococcus*, and *Sharpea* they constitute the family *Lactobacillaceae* within the order *Lactobacillales* of *Firmicutes*. Classification of lactobacilli is a subject of debate as phylogenomic analyses have revealed great inconsistencies within the current classification scheme (Claesson et al., 2008; Salvetti et al., 2012; Sun et al., 2015). These analyses indicate that the taxonomic status of family *Leuconostocaceae* and genus *Pediococcus* is dubious, as phylogenetic reconstructions show that most marker genes of these taxa branch within their corresponding *Lactobacillus* clusters. Therefore, we will consider *Lactobacillus*, *Pediococcus*, and *Leuconostocaceae* as a single phylogenetic unit and will refer to them as *Lactobacillus*. *Lactobacillus* strains play a major role in the production of a wide variety of fermented products. Others are naturally associated to mucosal surfaces of humans and animals and have been considered as probiotics (Tannock, 2004). Selected *Lactobacillus* species have the status of Generally Recognized as Safe or a Qualified Presumption of Safety, conferred by the Food and Drug Administration and European Food Safety Authority, respectively. However, the safety of each *Lactobacillus* strain intended to be used as probiotic needs to be assessed with specific studies, including antibiotic resistance profiling. A number of characteristics considered in the assessment of the safety of *Lactobacillus* species have already been reviewed (Bernardeau et al., 2008; Sanders et al., 2010).

*Bifidobacterium* are Gram-positive, anaerobic, obligate fermentative organisms, with a characteristic Y shape. *Bifidobacterium* species produce lactic acid and acetic acid from sugars at a 2:3 ratio (Pokusaeva et al., 2011). In addition to lactic acid production, bifidobacteria also share other phenotypic characteristics with lactobacilli. However, the analysis of their DNA sequences revealed that they are actually very distantly related to *Lactobacillus* as they are included in the phylum *Actinobacteria*. Bifidobacteria are commonly found as commensals of mammals, mostly colonizing the intestinal tract (Klijn et al., 2005; Bottacini et al., 2017). Bifidobacteria are one of the early colonizers of the gut of humans after birth, although their abundance will diminish following weaning. Since their first isolation by Tissier in 1899 from the feces of breast-fed infants, presence of bifidobacteria has been associated with a healthy microbiota leading to their commercialization in a number of fermented products or as food supplements.

Bifidobacteria and lactobacilli success in colonizing their natural habitats relies largely in their ability to utilize a variety of carbohydrates. Utilization of host-derived glycans by these bacteria has been addressed in numerous reviews [see for example, (Goh and Klaenhammer, 2015; Katayama, 2016; Bottacini et al., 2017; Turroni et al., 2018b)]. Comparative genomic analyses have also provided relevant information concerning the metabolic capabilities of bifidobacteria. A number of recent reviews have covered this topic and the readers are referred to them (Milani et al., 2016; Bottacini et al., 2017; Turroni et al., 2018a,b). Most of them have focused in oligosaccharide metabolism by bifidobacteria as information regarding glycans utilization by lactobacilli was scarce. However, recent studies have revealed that some lactobacilli can also use this source of carbohydrates and some of the metabolic pathways have been elucidated. As this is currently a very active research area, an update on the current knowledge on the genetic and metabolic aspects of host-derived glycan fermentation by bifidobacteria and lactobacilli is appropriate.

## Structural Characteristics of Free Human Milk Oligosaccharides (HMOs)

Human milk oligosaccharides are non-conjugated glycans composed of two to over twenty units with many structural isomers. From 100 to 200 different HMOs can be found in individual milk samples (Totten et al., 2012). So far, over 100 HMO structures have been determined (Kobata, 2010; Bode, 2012; Chen, 2015). HMOs constitute the third largest solid component of human milk after lactose and lipids (Kunz et al., 2000; Thurl et al., 2010). Concentration in mature human milk ranges from 10 to 15 g/l but relative proportions and global amounts of HMOs vary depending on the lactating stage, Lewis blood group and secretor status (Viverge et al., 1985; Kobata, 2010; Bode, 2012; Totten et al., 2012).

**FIGURE 1:**
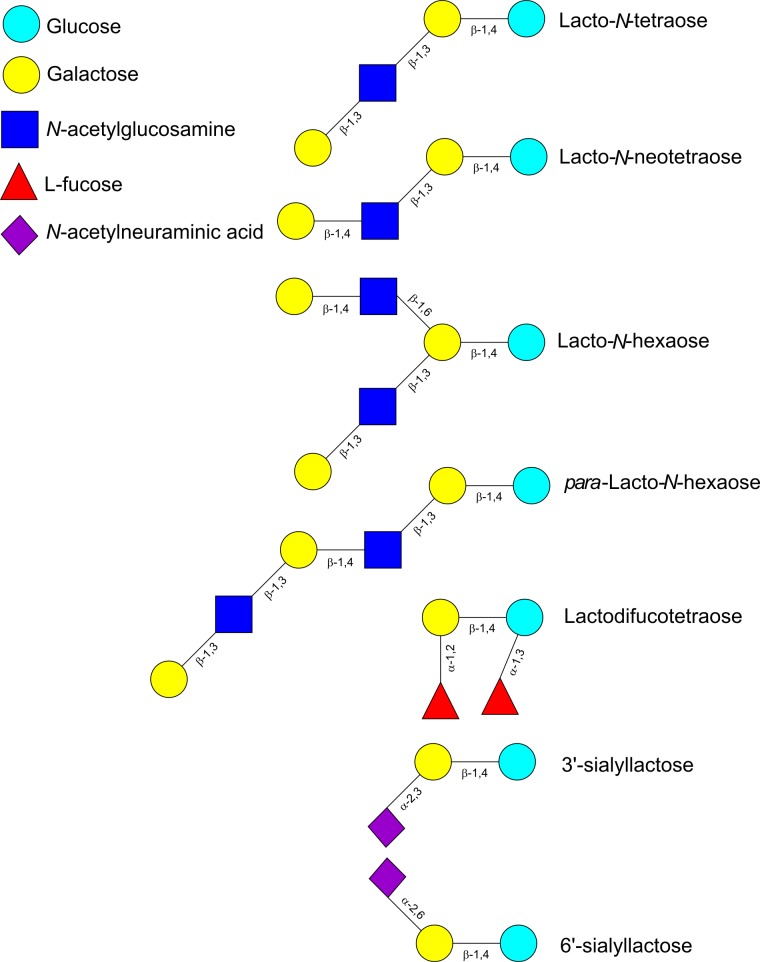
Schematic representation of selected oligosaccharides found in human milk.

Human milk oligosaccharides consist of combinations of five monosaccharide building blocks: D-glucose, D-galactose, *N*-acetylglucosamine (GlcNAc), L-fucose and sialic acid. The only form of sialic acid found in human milk is *N*-acetylneuraminic acid (Neu5Ac), while oligosaccharides present in milk of other mammals may contain *N*-glycolylneuraminic acid (Neu5Gc) too (Urashima et al., 2013). Almost all HMOs contain a lactose unit (Galβ1-4Glc) at their reducing end, that can be further elongated by the addition of β-1,3-linked lacto-*N*-biose (LNB; type-1 chain) or *N*-acetyllactosamine (LacNAc; type-2 chain) (**Table 1**). Elongation with LNB seems to terminate the chain, whereas LacNAc can be further extended (Bode, 2012; Chen, 2015) (**Figure 1**). Additionally, β-1,6 linkages introduce chain branching (**Figure 1**). LNB and LacNAc are also present as free sugars in human milk (Balogh et al., 2015). To these core structures as well as to lactose, fucosyl or sialyl residues can be added. Fucosyl residues can be added to galactose (via an α-1,2 linkage), glucose (α-1,3 linkage) or GlcNAc moieties (α-1,3 or α-1,4 linkages). Neu5Ac residues can be added to galactose (α-2,3 or α-2,6 linkages) or GlcNAc (α-2,6 linkage) (**Figure 1**) (Chen, 2015). Neu5Ac has a carboxyl group and therefore, sialylated HMOs contain one or more negative charges depending on the number of Neu5Ac residues added to the HMO backbone. About 70% of HMOs are fucosylated and about 20% are sialylated. The most abundant HMOs are lacto-*N*-tetraose (LNT), lacto-*N*-neotetraose (LNnT), as well as monofucosylated, monosialylated, difucosylated and disialylated lactose, LNT and LNnT (Kunz et al., 2000; Chen, 2015) (**Table 1**). Furthermore, HMOs containing the type-1 chain are more abundant than those containing a type-2 chain (Katayama, 2016). A few exceptions to these general structural features have also been identified such as HMOs containing a terminal *N*-acetylgalactosamine (GalNAc), 6-*O*-sulfo-monosaccharides or missing the glucose or lactose moiety at the reducing end (Chen, 2015).

## Utilization of HMOs by Bifidobacteria

Analyses of bifidobacterial genomes have highlighted the relevance of carbohydrate metabolism for these organisms as 14% of their genes encode proteins involved in carbohydrate metabolism (Milani et al., 2014; Turroni et al., 2018b). Particular *Bifidobacterium* species are specially adapted to exploit HMOs as a carbohydrate fermentable source and they carry in their genomes complete enzymatic machineries for their catabolism (Fushinobu, 2011; Garrido et al., 2013; Turroni et al., 2018b). Strains of *Bifidobacterium longum* subsp. *infantis* and *B. bifidum* grow very efficiently in laboratory culture media supplemented with HMOs, which are difficult to degrade by other bacterial groups (Asakuma et al., 2011). In fact, the genomes of these two organisms are enriched, compared to other *Bifidobacterium* species, in genes encoding glycosyl hydrolases essential for HMO and other host-glycan degradation (Turroni et al., 2018b). Genes required for glycan utilization often occur in clusters in bifidobacterial genomes as exemplified by the *B. longum* subsp. *infantis* gene cluster encoding glycosyl hydrolases and transporters required for the import and metabolism of HMOs (Sela et al., 2008). This 43-kb gene cluster encodes a variety of predicted or proven catabolic enzymes as well as extracellular solute binding proteins and permeases that are devoted to HMO metabolism (Sela et al., 2008; Milani et al., 2014). Utilization of HMOs requires the action of β-hexosaminidase, lacto-*N*-biosidase, β-galactosidase, fucosidase, and sialidase enzymes for complete degradation (**Table 3**). All these activities have been described in *Bifidobacterium* strains although the repertoire of enzymes and the utilization pathways vary from one species to another (Bottacini et al., 2017).

**Table 3:** Glycosidases from *Bifidobacterium* species with activity on host-derived glycans.

Name	Substrate	Linkage specificity	Species	Reference
**Sialidases**				
SiaBb2	3′-Sialyllactose	α-2,3; α-2,6	*B. bifidum*	Kiyohara et al., 2011,
	6′-Sialyllactose			2012
	*O*-Glycans			
	Gangliosides			
NanH1	Unknown	α-2,3; α-2,6	*B. infantis*	Sela et al., 2011
NanH2	Sialyllacto-*N*-tetraose	α-2,3; α-2,6	*B. infantis*	Sela et al., 2011
	Di-fucosylayed sialylated			
	lacto-*N*-hexaose-like			
	Disialyl-lacto-*N*-tetraose			
**α-Fucosidases**				
AfcA	2′-Fucosyllactose	α-1,2	*B. bifidum*	Katayama et al., 2004;
	Fucosyl-GM1			Sugiyama et al., 2017
AfcB	3′-Fucosyllactose	α-1,3; α-1,4	*B. bifidum*	Ashida et al., 2009
	Lacto-*N*-fucopentaose II			
Blon_0248	Lacto-*N*-fucopentaose III	α-1,3	*B. infantis*	Sela et al., 2012
Blon_0346	Fucα1-2Gal	α-1,2	*B. infantis*	Sela et al., 2012
Blon_0426	Lacto-*N*-fucopentaose III	α-1,3	*B. infantis*	Sela et al., 2012
Blon_2335	2′-Fucosyllactose	α-1,2; α-1,3	*B. infantis*	Sela et al., 2012
	3′-Fucosyllactose			
	Fucα1-2Gal			
	Lacto-*N*-fucopentaose I			
Blon_2336	3′-Fucosyllactose	α-1,2; α-1,3	*B. infantis*	Sela et al., 2012
	Lacto-*N*-fucopentaose III			
**Lacto-*N*-biosidases**				
LnbB	Lacto-*N*-tetraose	β-1,3	*B. bifidum*	Wada et al., 2008;
	Gb5; GA1			Gotoh et al., 2015
LnbX	Lacto-*N*-tetraose	β-1,3	*B. longum*	Sakurama et al., 2013;
	Lacto-*N*-fucopentaose I			Gotoh et al., 2015
	Sialyllacto-*N*-tetraose			
	Gb5; Globo H			
**Galactosyl-*N-*acetylhex- osamine phosphorylase**
LnpA	Lacto-*N*-biose	β-1,3	*B. longum*	Kitaoka et al., 2005;
	Galacto-*N*-biose		*B. breve*	James et al., 2016
**β-galactosidases**				
Bga42A (Blon_2016)	Lacto-*N*-tetraose	β-1,3	*B. infantis*	Yoshida et al., 2012
LntA	Lacto-*N*-tetraose	β-1,3; β-1,4	*B. breve*	James et al., 2016
	Lacto-*N*-neotetraose			
BglIII	Lacto-*N*-neotetraose	β-1,4	*B. bifidum*	Miwa et al., 2010
	*N*-Acetyllactosamine			
	Lacto-*N*-hexaose			
	*O*-Glycans			
Bga42A (Blon_2334)	Lacto-*N*-neotetraose	β-1,4	*B. infantis*	Yoshida et al., 2012
	Lactose			
	*N*-Acetyllactosamine			
**Exo-β-*N*-Acetylglucosaminidases**				
Blon_0459	Lacto-*N*-triose II	β-1,3; β-1,6	*B. infantis*	Garrido et al., 2012b
Blon_0732	Lacto-*N*-triose II	β-1,3; β-1,6	*B. infantis*	Garrido et al., 2012b
Blon_2355	Lacto-*N*-triose II	β-1,3	*B. infantis*	Garrido et al., 2012b
NahA	Lacto-*N*-triose II	β-1,3	*B. breve*	James et al., 2016
BbhI	Lacto-*N*-triose II	β-1,3	*B. bifidum*	Miwa et al., 2010;
	*O*-Glycans			Kiyohara et al., 2012
BbhII	*N*-Acetylglucosamine 6- sulfate *O*-linked glycans	β-1,6	*B. bifidum*	Miwa et al., 2010;
				Katoh et al., 2017
**Endo-β-*N*-Acetylglucosaminidase**				
EndoBI-1	*N*-Glycans	β-1,4	*B. infantis*	Garrido et al., 2012a
EndoBI-2	*N*-Glycans	β-1,4	*B. infantis*	Garrido et al., 2012a
EndoBB	*N*-Glycans	Unknown	*B. longum*	Garrido et al., 2012a
**α-*N*-Acetylglucosaminidases**				
AgnB	GlcNAcα1-4Gal	α-1,4	*B. bifidum*	Shimada et al., 2015
EngBF	Galb1-	α1Ser/Thr	*B. longum*	Fujita et al., 2005
	3GalNAcα1Ser/Thr			
NagBb	GalNAcα1Ser/Thr	α1Ser/Thr	*B. bifidum*	Kiyohara et al., 2012


*Fucosyl-GM1, Fucosyl-monosialoganglioside GM1; Fuc, fucose; Gal, galactose; Gb5, Gb5 pentasaccharide; Globo H, Globo H hexasaccharide; GlcNAc, N-acetylglucosamine; GalNAc, N-acetylgalactosamine*

Three different strategies of host-glycan utilization have been described in bifidobacteria. *B. longum* subsp. *infantis* encode ATP-binding cassette (ABC) transporters for internalization of intact oligosaccharides, which are subsequently degraded by intracellular glycosyl hydrolases (Garrido et al., 2011; Garrido et al., 2012a). Some strains of *B. breve* use a similar strategy (Ruiz-Moyano et al., 2013). In contrast, *B. bifidum* secretes a number of glycosyl hydrolases and takes up the resulting monosaccharide or disaccharide residues (Katayama et al., 2004; Wada et al., 2008; Ashida et al., 2009; Miwa et al., 2010; Turroni et al., 2010). A third strategy is used by “scavenger” bifidobacteria such as *B. breve* and *B. longum* subsp. *longum* which can only utilize a small fraction of HMOs, and sometimes only by taking advantage of other species such as *B. bifidum* that are capable of extracellular hydrolysis of larger HMOs (Bottacini et al., 2017).

### Trimming the Core Off: Sialidases and Fucosidases

#### Sialidases

As stated above, Neu5Ac residues can be added to the terminal positions of HMOs where their exposed location and charge prevent the action of several bacterial glycosidases. Removal of sialic acid from HMOs and intestinal glycoconjugates exposes the glycan moiety, which can then be catabolized (György et al., 1974). Sialidases have been identified in the genomes of some *Bifidobacterium* species such as *B. bifidum*, *B. breve*, *B. dentium*, *B. longum*, *B. mongoliense*, and *B. scardovii*. Bifidobacterial sialidases (EC 3.2.1.18) belong to glycoside hydrolase family 33 (GH33) according to the CAZy classification (Lombard et al., 2014).

Sialidase activity in bifidobacteria was first characterized in *B. bifidum* where a α-2,3 specific activity was described (Von Nicolai and Zilliken, 1972). In 2011, the cloning and characterization of the sialidase SiaBb2 of *B. bifidum* JCM1254 was published (Kiyohara et al., 2011). The analysis of the protein sequence revealed an N-terminal signal peptide and a C-terminal transmembrane region indicating that the enzyme is secreted and anchored to the membrane. Analysis of its specificity showed that it was active on α-2,3 and α-2,6 linkages although the activity on 6′-sialyllactose was only 4.45% of that on 3′-sialyllactose (Kiyohara et al., 2011). Cloning of the *siabb2* gene in *B. longum* 105-A enabled this strain to degrade sialylated oligosaccharides suggesting that SiaBb2 play a relevant role in the utilization of sialylated HMOs by *B. bifidum* (Kiyohara et al., 2011). Subsequent studies confirmed the extracellular location of SiaBb2 and its role in the assimilation of sialylated HMOs as a growth defect was observed in a *B. bifidum* JCM1254 *siabb2* defective mutant when grown with sialylated substrates (Nishiyama et al., 2017). Interestingly, this study also showed that SiaBb2 enhanced *B. bifidum* adhesion to mucosal surfaces via specific interactions with the α-2,6 linkage of sialyloligosaccharide and blood type A antigen on mucin carbohydrates (Nishiyama et al., 2017).

*Bifidobacterium longum* subsp. *infantis* ATCC 15697 harbors two sialidase encoding genes (*nanH1* and *nanH2*). Gene *nanH1* is located in a gene cluster dedicated to sialic acid catabolism whereas *nanH2* is located with a putative *N*-acetylneuraminate lyase (*nanA2*) within the 43 kb HMO cluster (Sela et al., 2011). The *nanH1* locus encodes an 83-kDa protein with a sialidase domain and a concanavalin A-like lectin domain that may facilitate substrate recognition and binding, whereas NanH2 is a 42 kDa protein that consists of a single sialidase domain (Sela et al., 2011). Both enzymes lack export signals, suggesting that they are intracellularly located. Their biochemical characterization showed that both enzymes cleaved α-2,3 and α-2,6-linked sialosides with preference for the α-2,6 linkage (Sela et al., 2011). However, when assayed with purified sialylated HMOs sialyllacto-*N*-tetraose and di-fucosylated sialylated lacto-*N*-hexaose-like oligosaccharide and, α-2,3- and α-2,6-linked disialyl-lacto-*N*-tetraose, NanH1 did not hydrolyze any of them, whereas NanH2 rapidly degraded them (Sela et al., 2011). Consistently with this observation, expression of NanH1 was not induced by HMOs while expression of NanH2 was (Sela et al., 2011). The authors of the study suggested that NanH1 may be active on other sialylated substrates (Sela et al., 2011) but so far, they have not been identified.

#### Fucosidases

α-fucosidases catalyze the release of α-linked fucose residues. Most fucosidases harbored by bifidobacteria belong to CAZY families 29 or 95 (GH29 and GH95). The first bifidobacterial cloned and characterized fucosidase was AfcA from *B. bifidum*, a extracellular cell-anchored α-1,2-specific fucosidase (Katayama et al., 2004). AfcA has 1959 amino acids divided among three domains: an N-terminal domain with unknown function, a catalytic domain, and a C-terminal bacterial Ig-like domain. AfcA was the first characterized GH95 protein (Katayama et al., 2004). GH29 and GH95 enzymes differ in their mechanism of action. GH29 members possess a double displacement mechanism that relies on two carboxyl groups. In the first step of catalysis, one of the carboxyl groups acts as a general acid, assisting aglycone departure, whereas the second carboxyl group attacks the anomeric carbon, generating a covalent glycosyl-enzyme intermediate. In the second step, the first carboxyl group acts as base, activating an incoming water molecule for nucleophilic attack on the anomeric carbon of the glycosyl-enzyme intermediate (Sulzenbacher et al., 2004). GH95 enzymes rely in a unique inverting mechanism where a water molecule in the active center establishes hydrogen bonds with two Asn residues thus being activated for a nucleophilic attack on the C1 atom of L-fucose. On the other hand, a Glu residue acts as a proton donor for the O_2_ atom to aid the release of products (Katayama et al., 2004; Nagae et al., 2007). Subsequent studies identified a second α-fucosidase in *B. bifidum*, AfcB, a 1493-amino acid extracellular cell-anchored polypeptide belonging to the GH29 family (Ashida et al., 2009). AfcB releases α-1,3- and α-1,4-linked fucosyl residues. Together, both enzymes enable *B. bifidum* the removal of all fucosyl residues in HMOs.

Other bifidobacterial strains also encode several α-fucosidases (Bunesova et al., 2016; Garrido et al., 2016). *B. longum* subsp. *infantis* ATCC 15697 harbors five α-fucosidases: Blon_0248 (GH29), Blon_0426 (GH29), Blon_2335 (GH95), Blon_2336 (GH29), and Blon_0346 (GH151) (Sela et al., 2012). The last one possesses a pfam01120 α-fucosidase domain at the 5′ terminus, a middle β-galactosidase trimerization domain (pfam08532), which suggests that this enzyme may be active as a trimer, and it is quite divergent from other α-fucosidases (Sela et al., 2012). Paralogs Blon_0248 and Blon_0426 are 95% identical (Sela et al., 2012). Blon_2335 and Blon_2336 are located in the HMO utilization gene cluster (Sela et al., 2012). Blon_2336 has a 33% amino acid identity to the fucosidase domain of AfcB although, it lacks secretory signals and cell envelope anchoring signatures of AfcB indicating that it is an intracellular enzyme (Sela et al., 2012). Blon_2335 shares 26% amino acid identity along its alignable region to AcfA, although it has a shorter sequence, which corresponded solely to the AfcA GH95 catalytic domain (Sela et al., 2012). The activity of *B. longum* subsp. *infantis* ATCC 15697 α-fucosidases has been characterized. Blon_0248 and Blon_0426 cleaved α-1,3 linkages. Blon_2335 cleaved α-1,2 linkages and also displayed some activity on α-1,3, whereas Blon_2336 preferentially cleaved α-1,3 linkages and to a lesser extent α-1,2 linkages. Finally, Blon_0346 only displayed some activity on Fucα1-2Gal whereas it did not display any activity on 2′-fucosyllactose or 3′-fucosyllactose (Sela et al., 2012).

### Breaking up the Core: Bifidobacterial Pathways for Utilization of Core HMOs

Removal of fucosyl and sialyl residues is most times required for further utilization of HMOs by bifidobacteria. The cores of HMOs are then subjected to the action of different glycosidases. The pathways of degradation of core HMOs to their constituent monosaccharides vary from one species to other, although the pathways of monosaccharide utilization are often common to all of them. Among HMOs, the pathways of utilization of LNT and LNnT are the best characterized.

### Utilization of Lacto-*N*-Tetraose

#### Lacto-*N*-Biosidase Pathway

**FIGURE 2:**
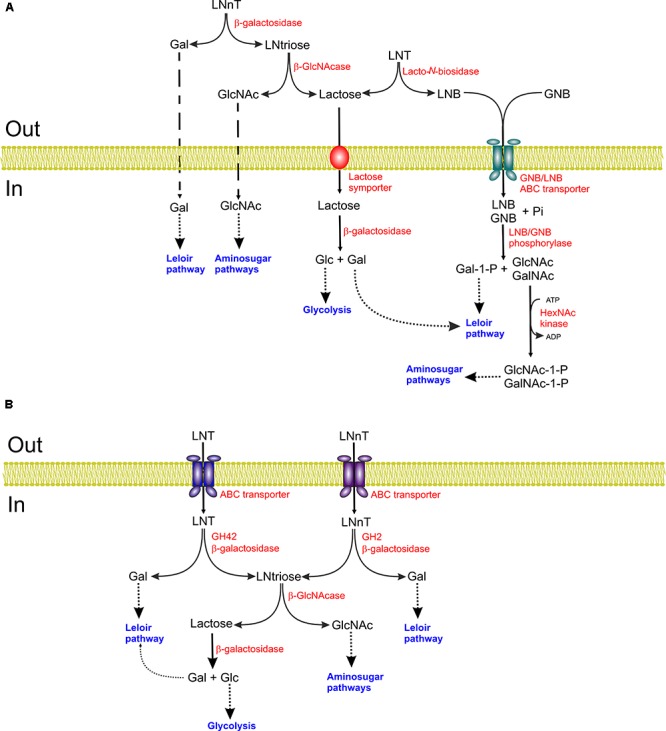
Schematic representation of the lacto-*N*-tetraose (LNT) and lacto-*N*-neotetraose (LNnT) catabolic pathways of *Bifidobacterium bifidum* **(A)** and *B. longum* subsp. *infantis* **(B)**. Gal, galactose; LNtriose, lacto-*N*-triose II; LNB, lacto-*N*-biose; GNB, galacto-*N*-biose; GlcNAc, *N*-acetylglucosamine; Glc, glucose; GalNAc, *N*-acetylgalactosamine.

Lacto-*N*-biosidase (LNBase; EC 3.2.1.140) is an endoglycosidase that brakes non-terminal β-1,3 linkages between GlcNAc and galactose in type-1 HMOs releasing LNB. In the case of LNT, the action of LNBase produces LNB and lactose (**Figure 2**). LNBase activity has been detected in strains of *B. bifidum* and *B. longum*, but not in *B. breve* or *B. catenulatum* (Wada et al., 2008). A LNBase encoding gene, *lnbB*, was first cloned and characterized in *B. bifidum* JCM1254 (Wada et al., 2008). The gene encoded a protein of 1112 amino acid residues with a predicted molecular mass of 120 kDa. The deduced amino acid sequence contained a signal peptide and a membrane anchor at the N-terminal and C-terminal ends, respectively, indicating an extracellular location. Analysis of conserved domains revealed the presence of a glycosyl hydrolase family 20 (GH20) domain (aa 179–496), a carbohydrate-binding module 32 (CBM32; aa 784–932), and a bacterial immunoglobulin (Ig)-like 2 domain (aa 960–1041) (Wada et al., 2008). The enzyme was active on LNT but not on fucosylated forms of LNT or on LNnT. It was not active on α-linked LNB derivatives, thus indicating that it acts preferentially on unmodified β-linked LNB (Wada et al., 2008). The determination of the structure of the active core of the enzyme (aa 41–663) provided evidence about the specificity of *B. bifidum* lacto-*N*-biosidase (Ito et al., 2013). The structure displayed three domains: an N-terminal domain (N-domain, residues 41–178); a catalytic (β/α)_8_ barrel domain (barrel domain, residues 179–496), and a C-terminal domain (C-domain, residues 497–662). The active site is located at the center of the barrel domain and it was observed that all the hydroxyl groups of LNB form hydrogen bonds with the surrounding amino acids (Ito et al., 2013). These extensive interactions account for the specificity of the enzyme. The conformation of the active site also accounts for the lack of activity on fucosylated glycans as no space for accommodating a fucosyl residue is present (Ito et al., 2013). The analysis of the reaction mechanism confirmed a retaining mechanism as expected for GH20 hydrolases.

Screening of a genomic library of *B. longum* JCM1217 allowed the identification of a LNBase in this organism (Sakurama et al., 2013). The shortest fragment conferring LNBase activity comprised two genes, *lnbX* (1599 aa) and *lnbY* (280 aa). Remarkably, they did not share sequence similarities with any characterized proteins. Analysis of the sequences revealed the presence of a signal peptide and a membrane anchor in *lnbX* and, a signal peptide in *lnbY*. The biochemical characterization of these proteins showed that LnbX constitute the active enzyme whereas LnbY is required for proper folding of LnbX (Ito et al., 2013). In contrast to *B. bifidum* LnbB, LnbX was active on lacto-*N*-fucopentaose I and sialyllacto-*N*-tetraose (Sakurama et al., 2013).

Both LnbB and LnbX can also release galacto-*N*-biose (GNB) (**Table 2**) from some oligosaccharides although with lower specific activities than LNB (Gotoh et al., 2015). Both enzymes could hydrolyze 4-nitrophenyl-β-GNB, but not α-linked GNB. LnbX also hydrolyzed the Gb5 pentasaccharide and the Globo H hexasaccharide, whereas LnbB was active on Gb5 and the GA1 tetrasaccharide (**Table 2**) (Gotoh et al., 2015). Therefore, as previously observed for LNB-containing oligosaccharides, LnbX hydrolyzes fucosylated derivatives whereas LnbB does not.

Lacto-*N*-biose and GNB can be metabolized by the products of the LNB-GNB gene cluster, which is present in *B. bifidum*, *B. breve*, *B. longum* subsp. *Infantis*, and *B. longum* subsp. *longum*. The identification of the 1,3-β-galactosyl-*N*-acetylhexosamine phosphorylase in *B. bifidum* (Derensy-Dron et al., 1999) paved the way for the elucidation of this pathway. The enzyme catalyzes the phosphorolytic cleavage of the β-glycosidic linkage of LNB or GNB releasing galactose-1-P and GlcNAc or GalNAc, respectively (Derensy-Dron et al., 1999). The encoding gene, *lnpA*, was first cloned from *B. longum* JCM1217 (Kitaoka et al., 2005). This gene was located in a seven gene operon also encoding an ABC transporter (*gltA, gltB, gltC*), a *N*-acetylhexosamine 1-kinase (*nahK*) (Nishimoto and Kitaoka, 2007), an UDP-glucose-hexose 1-phosphate uridylyl transferase (*lnpC*) and an UDP-glucose/GlcNAc 4-epimerase (*lnpD*) (**Figure 2**). Analysis of GltA has shown that it is a solute-binding protein, which binds GNB, LNB, and also LNT albeit with a higher *K*_d_ (Suzuki et al., 2008; Kitaoka, 2012) thus, supporting the role of GltABC as a GNB/LNB transporter. LnpA subsequently cleaves internalized LNB or GNB into galactose-1-P and GlcNAc or GalNAc, as previously indicated. Galactose-1-P is transformed into α-glucose-1-P by the concerted action of LnpC and LnpD. LnpC, a GalT (EC 2.7.7.12) type enzyme, transfers UMP from UDP-glucose to Gal-1-P resulting in the production of glucose-1-P and UDP-Gal, the latter being epimerized to UDP-glucose by LnpD, a GalE (EC 5.1.3.2) type enzyme (Nishimoto and Kitaoka, 2007). GalNAc or GlcNAc on the other hand are phosphorylated to GalNAc-1-P or GlcNAc-1-P by LnpB (Nishimoto and Kitaoka, 2007). The resulting products are substrates of LnpC and LnpD, which have the unusual property of acting also on GalNAc-1-P and GlcNAc-1-P (Nishimoto and Kitaoka, 2007). The resulting products, glucose-1-P and GlcNAc-1-P then enter the glycolytic and amino sugar pathways, respectively (Wada et al., 2008; Kitaoka, 2012). This pathway has also been characterized in *B. breve* (James et al., 2016).

### β-Galactosidase Pathway

*Bifidobacterium longum* subsp. *infantis* ATCC15697 utilizes a different pathway for LNT degradation. In this organism, LNT is internalized by an ABC transporter (Garrido et al., 2011) and hydrolyzed by a specific GH42 β-galactosidase (Bga42A; locus tag Blon_2016) that releases galactose and lacto-*N*-triose II (GlcNAcβ1-3Galβ1-4Glc) which is subsequently degraded by β-*N*-acetylglucosaminidase to GlcNAc and lactose (Yoshida et al., 2012) (**Figure 2**). Bga42A is specific for LNT as its activity was very weak on LNB and the characterization of other β-galactosidases encoded by this organism indicated that it is the only one acting on type-1 HMOs (Yoshida et al., 2012). *B. longum* subsp. *infantis* ATCC15697 encode three putative GH20 intracellular β-*N*-acetylglucosaminidases. (Blon_0459, Blon_0732 and Blon_2355). Their biochemical analysis showed that all of them were active on GlcNAc β-1,3-linked and two of them, Blon_0459 and Blon_0732, also cleaved GlcNAc β-1,6-linked, whereas none of them were active on β-linked GalNAc, glucose or galactose (Garrido et al., 2012b). All of them were active on lacto-*N*-triose II and could also cleave the β-1,3 linkage of lacto-*N*-hexaose (LNH) after removal of the terminal β-1,3-linked galactose residue of this molecule. Furthermore, removal of the terminal β-1,4-linked galactose residue allowed the cleavage of the β-1,6 linkage by Blon_0732 and, to a lesser extent, Blon_0459 (Garrido et al., 2012b). In this way, the hydrolysis of LNT or LNH results into lactose, that can be cleaved by a β-galactosidase, galactose and GlcNAc. The pathway of *B. breve* UCC2003 for utilization of LNT has also been elucidated. As *B. longum* subsp. *infantis*, this organism also takes up LNT that is sequentially degraded by the β-galactosidase LntA and the *N*-acetylglucosaminidase NahA (James et al., 2016).

### Utilization of Lacto-*N*-Neotetraose

*Bifidobacterium bifidum* metabolizes extracellularly LNnT by the sequential action of an extracellular β-galactosidase that releases galactose and lacto-*N*-triose II which in turn is the substrate of a β-*N*-acetylglucosaminidase that produces GlcNAc and lactose (**Figure 2**) (Miwa et al., 2010). Characterization of four β-galactosidases encoded by *B. bifidum* NCIMB41171 (BbgI, BbgII, BbgIII, and BbgIV), revealed that BbgI and BbgIV were most active on lactose, BbgII on β-D-1,6-galactobiose and BbgIII on LacNAc (Goulas et al., 2009). In a different study, among five β-galactosidases encoded by *B. bifidum* JCM1254, β-galactosidase BbgIII was identified as an extracellular membrane-anchored multidomain glycosidase belonging to GH2 family. BbgIII acts preferentially on the β-1,4 linkage of LNnT and LacNAc and it cannot hydrolyze fucosylated derivatives of these glycans (Miwa et al., 2010). BbgIII can also act, although less efficiently, on the branched HMO lacto-*N*-hexaose (Miwa et al., 2010). In the same study, three putative GH20 β-*N*-acetylhexosaminidases were identified, two of them predicted to be membrane-anchored. The latter two, BbhI and BbhII, were characterized. BbhI hydrolyzed lacto-*N*-triose II but not LNT or LNnT, indicating that it is an exo-acting enzyme (Miwa et al., 2010). In contrast, BbhII had very weak activity on lacto-*N*-triose II but it was more active on GlcNAc β-1,6-linked, which led to the suggestion that BbhII may participate in the degradation of glycans containing β-1,6 linkages, such as lacto-*N*-hexaose, in cooperation con BbhI (Miwa et al., 2010). In summary, the concerted action of BbgIII and BbhI degrades LNnT to galactose, GlcNAc and lactose that can then be internalized and channeled to the glycolytic pathway.

*Bifidobacterium longum* subsp. *infantis* ATCC15697 utilizes the same strategy for LNnT degradation as for LNT (see above) albeit it uses a different β-galactosidase (**Figure 2**) (Yoshida et al., 2012). As LNT, LNnT is internalized and hydrolyzed into galactose and lacto-*N*-triose II by Bga2A, a GH2 β-galactosidase whose encoding gene (Blon_2334) is located in the HMO gene cluster. This enzyme is most active on lactose but it also degrades efficiently LacNAc and LNnT but did not hydrolyzes LNT (Yoshida et al., 2012). *B. breve* UCC2003 also internalizes LNnT and degrades it by the sequential action of β-galactosidases and the *N*-acetylglucosaminidase NahA (James et al., 2016). In contrast to LNT, which is exclusively cleaved by LntA (see above), in *B. breve* LNnT can be degraded by LntA and other β-galactosidases, as a LntA-defective mutant was unable to grow on LNT but it did so on LNnT (James et al., 2016).

In summary, bifidobacterial pathways of HMOs utilization are relatively well characterized in species that colonize the human infant gut. However, transport of HMOs or HMO-derived monosaccharides or disaccharides will require further effort. So far, very few studies have addressed this problem. In part, the analysis of HMOs transport may be complicated by the presence of multiple putative transporters that may be involved in HMO uptake. The study carried out by Garrido et al. (2011) indicated that some of the *B. longum* subsp. *infantis* ABC transporters possibly have overlapping functions. The study of James et al. (2016) in *B. breve* UCC2003 also pointed to overlapping activities of some transporters at least for some HMOs. Regulation of the expression of genes involved in HMO utilization also remains largely unexplored. The recent study of James et al. has addressed this issue in *B. breve* UCC2003 (James et al., 2018) but information on other model bifidobacteria such as *B. bifidum* and *B. longum* subsp. *infantis* is still very scarce.

## Utilization of Hmos by Lactobacilli

Unlike bifidobacteria, the use of HMOs by members of the genus *Lactobacillus* is a relatively unexplored field and the capacity to use this carbon source appears rather limited. The genomes of intestinal lactobacilli generally carry a large set of glycosyl hydrolases (Pridmore et al., 2004; Altermann et al., 2005; Azcarate-Peril et al., 2008), but they are probably responsible for the exploitation of carbohydrates derived from the diet and many characterized sugar transporters are involved in the transport of mono and disaccharides not necessarily related to host glycans, with the exception of GlcNAc. Lactobacilli possess phosphoenolpyruvate-dependent: sugar phosphotransferase systems (PTS) for the internalization of this monosaccharide, which is abundant in host mucosae, and the corresponding encoding genes are induced in the intestinal tract of animal models (Marco et al., 2007; Golomb et al., 2016). The capacity to hydrolyze terminal sugars from HMOs is also reduced in lactobacilli. Fucosidases are restricted to few species (see below) and sialidases have not been described for this group, although a report exits on the use 6′-sialyllactose HMO by *Lactobacillus delbrueckii* ATCC7830 (Ito et al., 2013). Nevertheless, clusters for sialic acid catabolism are found in some species. In *L. sakei* 23K two adjacent and divergently transcribed gene clusters, *nanTEAR* and *nanKMP*, are present which contain genes involved in the catabolism of Neu5Ac (Anba-Mondoloni et al., 2013). The *nanT* gene codes for a Na^+^/Neu5Ac symporter, while internalized Neu5Ac is catabolized via a *N*-acetylneuraminic acid lyase, a *N*-acetylmannosamine kinase and a *N*-acetylmannosamine-6-P epimerase encoded by the products of *nanA*, *nanK*, and *nanE*, respectively, yielding GlcNAc-6-P which is channeled via glycolysis. The role of *nanM* and *nanP* is not known. NanM is similar to putative sugar-phosphate isomerases/epimerases and it is also present in other *nan* clusters in lactobacilli. NanP is a protein with putative transmembrane segments that resembles a Na^+^/H^+^ antiporter. This protein could work in the regulation of internal pH after Neu5Ac uptake and metabolism or in exporting Na^+^ internalized with Neu5Ac via NanT. Accordingly, an *L. sakei nanP* mutant is unable to use Neu5Ac (Anba-Mondoloni et al., 2013). *L. sakei* is a species adapted to grow in meat products, where the use of Neu5Ac may have an impact in the capacity to colonize this niche. It is known that Neu5Ac utilization provides intestinal bacteria with a competitive advantage at the GI tract (McDonald et al., 2016) and *nan* clusters have been described in other intestinal lactobacilli (*L. plantarum*, *L. salivarius*, and *L. paracasei*) (Almagro-Moreno and Boyd, 2009; Hammer et al., 2017), although Neu5Ac catabolism is not exclusive to this group and *nan* clusters can be found in lactobacilli isolated from other niches (see: Microbial Genome Database^1^).

### Fucosidases in Lactobacilli and the Use of Fucosyloligosaccharides

So far, only three α-L-fucosidases (AlfA, AlfB, and AlfC) from the GH29 family have been characterized in lactobacilli (**Table 4**) (Rodríguez-Díaz et al., 2011). These enzymes are mostly present in strains of the phylogenetically related group *L. casei–paracasei–rhamnosus* with different strains carrying one, two or the complete set of enzymes. All three enzymes are intracellular homotetramers (Rodríguez-Díaz et al., 2011) that depend on the transport of fucosylated carbohydrates for their function. No specific substrates have been identified so far for AlfA, which is able to release α1,6-linked fucose residues from some oligosaccharides albeit with low efficiency (Rodríguez-Díaz et al., 2011). On the other hand, AlfB and AlfC possess high activity on α-1,3 and α-1,6 bonds in fucosyl-GlcNAc, respectively (Rodríguez-Díaz et al., 2011). Fucosylα1-3GlcNAc (3FN) is part of the Lewis X antigen, present in glycoproteins at the mucosa, whereas fucosylα1-6GlcNAc (6FN) is found in the core of *N*-glycosylation (fucosylα1-6GlcNAc-asparagine). Contrarily to the α-L-fucosidases characterized in bifidobacteria, the *L. casei* enzymes are highly specific for α1,3 and α1,6 linkages, respectively. Furthermore, these enzymes act on disaccharides rather than other long oligosaccharides containing these bonds. This, together with the fact that they are intracellular enzymes, supports the idea that *L. casei* is specialized in transport and metabolism of short fucosyl-oligosaccharides. This observation suggests that *L. casei* would follow a “scavenger” strategy similar to that of *B. breve* and *B. longum* subsp. *longum*, which use short host-derived glycans that are probably released by the hydrolytic activities of other members of the microbiota.

**Table 4:** Glycosidases from *Lactobacillus casei* with activity on host-derived glycans.

Type	Name	Substrate	Linkage specificity	Reference
α-Fucosidase	AlfA	Unknown	α-1,6	Rodríguez-Díaz et al., 2011
α-Fucosidase	AlfB	Fucosylα1-3GlcNAc	α-1,3	Rodríguez-Díaz et al., 2011
α-Fucosidase	AlfC	Fucosylα1-6GlcNAc	α-1,6	Rodríguez-Díaz et al., 2011
Phospho-β-galactosidase	GnbG	Lacto-*N*-biose 6- phosphate	β-1,3	Bidart et al., 2014
		Galacto-*N*-biose 6- phosphate		
β-*N*-Acetylglucosaminidase	BnaG	Lacto-*N*-triose II	β-1,3	Bidart et al., 2016
Phospho-β-galactosidase	LacG	*N*-Acetyllactosamine 6-phosphate	β-1,4	Bidart et al., 2018
		Lactose 6-phosphate		


**FIGURE 3:**
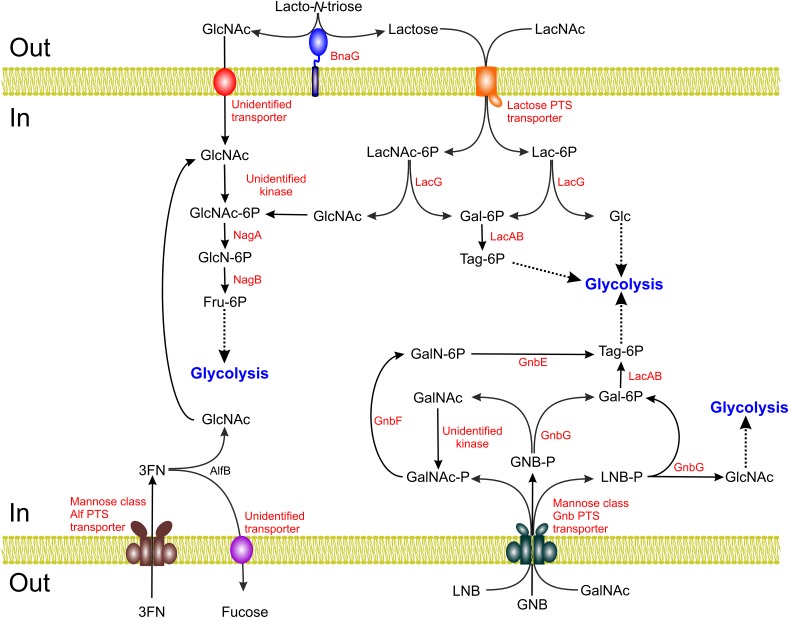
Schematic representation of the lacto-*N*-biose (LNB), galacto-*N*-biose (GNB), *N*-acetyllactosamine (LacNAc), lacto-*N*-triose II and fucosyl-α1,3-*N*-aceylglucosamine (3FN) catabolic pathways of *Lactobacillus casei*. Gal, galactose; Lac, lactose; GlcNAc, *N*-acetylglucosamine; GlcN, glucosamine; Fru, fructose; Glc, glucose; Tag, tagatose; GalNAc, *N*-acetylgalactosamine.

Gene *alfB* is clustered with the transcriptional regulator alfR and, divergently transcribed, genes *alfEFG* encoding a mannose-class PTS transporter (**Figure 3**). The expression of this cluster is induced in the presence of 3FN by a mechanism mediated by the transcriptional repressor AlfR (Rodriguez-Diaz et al., 2012). Remarkably, transport of 3FN via this PTS is not coupled to phosphorylation, which fits with the fact that the sugar position phosphorylated via the mannose-class PTSs (carbon-6) does not contain a phosphorylatable hydroxyl group in L-fucose (6-deoxy-galactose).

The fate of the released L-fucose varies depending on the species. Most *L. casei/paracasei* strains do not possess the capacity to metabolize the intracellularly generated L-fucose and they expel it quantitatively to the growth medium when growing on fucose-containing sugars (Rodriguez-Diaz et al., 2012). In most strains of *L. rhamnosus*, the presence of L-fucose metabolizing enzymes supports the metabolism of this monosaccharide. The genes for L-fucose uptake and metabolism have been characterized in *L. rhamnosus* GG. Contrarily to bifidobacteria, the L-fucose catabolic route in lactobacilli is similar to that described for *Escherichia coli* and some intestinal bacteria such as species of *Bacteroides* (Becerra et al., 2015). L-fucose is transported by a sugar permease belonging to the major facilitator superfamily before being converted to L-fuculose. Successive enzymatic steps phosphorylate L-fuculose and split the resultant compound into L-lactaldehyde and dihydroxyacetone phosphate, which is channeled via glycolysis. Depending on the redox conditions L-lactaldehyde can be metabolized to L-lactate or L-1,2-propanediol (Becerra et al., 2015).

### Metabolism of Type I HMOs Core Lacto-*N*-Biose and the Structurally Related Disaccharide Galacto-*N*-Biose

In *L. casei* the GnbG enzyme encodes a GH35 glycosidase with high specificity for β-1,3 linkages that is able to hydrolyze LNB as well as GNB (Bidart et al., 2014) (**Figure 3**). This enzyme is clustered in the *gnb* operon which also encodes a mannose-class PTS involved in the transport and phosphorylation of these two disaccharides (PTS^Gnb^). GNB-P and LNB-P resulting from the concomitant transport and phosphorylation of GNB and LNB via the PTS^Gnb^ might be the natural substrates of GnbG as this enzyme displayed low activity on their non-phosphorylated forms. The *gnb* operon also carries two enzymes for the metabolism of GalNAc: *gnbE*, encoding galactosamine 6-phosphate isomerizing deaminase and *gnbF*, encoding GalNAc 6-phosphate deacetylase. Several facts suggest that the *gnb* operon is specialized in GNB metabolism: (i) the absence of GnbE and GnbF only impairs growth on GNB but not on LNB; (ii) the *gnb* genes are highly induced during growth on GNB and GalNAc while almost no induction occurred with LNB and (iii) the PTS^Gnb^ is also required for growth on GalNAc, but not on GlcNAc (Bidart et al., 2014). Thus, structural similarities between GNB and LNB that allow transport of LNB through the PTS^Gnb^ and its subsequent hydrolysis by GnbG seem responsible for LNB utilization. GNB is part of the core of *O*-glycosylation present in glycoproteins such as mucin (see below). The *gnb* gene cluster is present in strains of the closely related species *L. casei–paracasei–rhamnosus*. These disaccharides are also metabolized by other species of intestinal lactobacilli (*L. gasseri* and *L. johnsonii*) although homologs of the *gnb* genes are not detected in their chromosomes, suggesting that as yet undiscovered pathways for the use of these mucosal-derived disaccharides are active in these bacteria (Bidart et al., 2014, 2017).

### Utilization of Other Core HMOs in Lactobacilli: Lacto-*N*-Triose II

The utilization of lacto-*N*-triose II by *L. casei* relies on the activity of the β-*N*-acetylglucosaminidase BnaG (GH20) (**Figure 3**). This protein is a cell-wall protein covalently bound to peptidoglycan via a sortase-dependent LPXTG motif present in its C-terminal portion. BnaG cleaves lacto-*N*-triose II into GlcNAc and lactose which can be subsequently transported and metabolized (Bidart et al., 2016). The enzyme is also active on 3′-*N*-acetylglucosaminyl-mannose and 3′-*N*-acetylgalactosaminyl-galactose, two sugars present in glycoproteins and glycosphingolipids, but it shows very low activity on other β-1,3 bonds such as those present in LNB and GNB (Bidart et al., 2016). However, BnaG is not involved in the metabolism of 3′-*N*-acetylglucosaminyl-mannose (Bidart et al., 2014), indicating that *L. casei* must possess other as yet unidentified β-1,3 glycosidases acting on this sugar. Furthermore, these bacteria lack glycosidases acting on LNT or LNnT that would release lacto-*N*-triose II from LNT or LNnT and are therefore unable to grow on these HMOs. Homologs of *bnaG* are common in strains of the *L. casei*–*paracasei*–*rhamnosus* group and are located upstream from the *gnb* operon together with *manA*, which codes for a mannose 6-phosphate isomerase. However, *bnaG* and *manA* do not follow the same regulation as the *gnb* genes: their expression is not inducible by GNB nor lacto-*N*-triose II (Bidart et al., 2016), although it is subject to carbon catabolite repression (Monedero et al., 1997).

### The Versatility of the Lactose-Utilization System: Metabolism of *N*-Acetyllactosamine

Owing to its economical relevance, lactose metabolism in lactic acid bacteria such as *Lactococcus*, *Streptococcus*, and *Lactobacillus* has been the subject of an important research effort in the past. Different lactose uptake systems (symporters, antiporters, ABC transporters, and PTSs) and β-galactosidases belonging to different families have been characterized at the genetic and biochemical level (de Vos and Vaughan, 1994). Genes involved in lactose metabolism can be located in lactobacilli at their chromosomes or as part of mobile genetic elements such as plasmids. It was assumed that *lac* systems were specific for lactose utilization until the recent discovery that the metabolism of LacNAc, the type 2 HMO building block, is dependent in *L. casei* on its *lac* genes (Bidart et al., 2018) (**Figure 3**). LacNAc is also present as a constituent of the ABO and Lewis blood group antigens, which are found in red blood cells but are also expressed as part of mucosal secretions and *N*- and *O*-glycans and glycolipids, playing a relevant role in the intestinal ecology (Stanley and Cummings, 2009). Growth of *L. casei* in LacNAc is as efficient as growth in lactose in terms of specific growth rate and final biomass and, compared to lactose, LacNAc is a stronger inducer of *lac* genes. Lactose and LacNAc are transported and phosphorylated by a specific PTS [PTS^lac^; encoded by *lacE* and *lacF* genes (Gosalbes et al., 1999)] and hydrolyzed into galactose 6-phosphate and glucose/GlcNAc by the product of the *lacG* gene. LacG is a 55 kDa protein belonging to the GH1 family, which comprises glycosidases as well as phospho-glycosidases, which act on phosphorylated disaccharides. Other characterized GH1 β-glycosidases from bifidobacteria are able to act on lactose and LacNAc (e.g., the *B. bifidum* BbgI, BbgIII, and BbgIV enzymes) but LacG is completely unable to hydrolyze these disaccharides. This observation supports the idea that phosphorylated LacNAc and lactose, the products of transport through the PTS^lac^, are the natural substrates of this enzyme. Thus, mutants defective in the enzyme that channels the galactose 6-phosphate resulting from lactose 6-phosphate or LacNAc 6-phosphate hydrolysis by LacG toward the lower part of glycolysis, the galactose 6-phosphate isomerase (EC 5.3.1.26) from the tagatose 6-phosphate pathway, display a reduced growth in LacNAc (Bidart et al., 2018).

The characteristics of the *L. casei lac* system support the hypothesis that it may have evolved primarily as a LacNAc utilization system, but its capacity to metabolize other disaccharides with β-1,4 configuration, such as lactose, was exploited later to grow in milk. A similar situation has been described for the well-known *E. coli lac* system, which is believed to primarily serve for the utilization of β-galactosyl glycerol derived from plant material at the intestinal tract, instead of lactose (Egel, 1988). Lactose utilization systems similar to that of *L. casei* are widespread in some *Lactobacillus* species and their capacity to metabolize other substrates different to lactose deserve further studies.

## Conjugated Glycans: Glycoproteins and Glycolipids

### Glycan Structures in Mucins

The GI epithelium is covered by a thick protective layer of mucus that plays crucial roles in the symbiotic relationships established between the host and the intestinal microbiota. Mucus is mainly composed by mucins, which are encoded by more than 20 genes in the human genome and can be secreted or membrane attached (Bansil and Turner, 2018). The expression of different mucin types differs along the GI tract with MUC2 representing the major soluble and gel-forming mucin at the small intestine and colon (Moran et al., 2011). Mucins are characterized for being highly glycosylated (carbohydrates represent 50–80% w/w), with a very large proportion of *O*-glycosylation modifications. Mucins contain domains characterized by tandem repeats of the amino acids proline, threonine, and serine (PTS domains) which represent targets for *O*-glycosylation. In this type of modification a bond is formed between the amino acids Ser/Thr and GalNAc that is extended with residues of galactose, GalNAc and GlcNAc from four main cores: type 1, type 2, type 3, and type 4 (**Table 2**). The resulting polysaccharide structures can be modified by the addition of ABO and Lewis blood groups (Rossez et al., 2012), and they can be terminally fucosylated, sialylated and sulfated. *N*-glycosylation in mucins is less abundant, but it is a common modification in mucosal and human milk proteins. This modification takes place at a consensus protein sequence (AsnXxxSer/Thr/Cys, Xxx is any amino acid), where a core of *N*,*N*′-diacetylchitobiose disaccharide (ChbNAc) is covalently bound to the asparagine at the consensus sequence and the basal GlcNAc can be fucosylated via an α-1,6 bond. *N*-glycosylation in mucins and milk proteins is also elongated by the so-called multiantennary complex, high mannose or hybrid oligosaccharide structures carrying mannose, galactose and GlcNAc, which can be fucosylated and sialylated (Stanley and Cummings, 2009; Karav et al., 2017). Thus, *O-* and *N*-glycosylated proteins, especially mucins, constitute a carbon and energy source for intestinal bacteria in the form of oligosaccharides (Sicard et al., 2017). Genera and species particularly adapted to this niche, such as *Bacteroides*, *Ruminococcus* or *Akkermansia muciniphila*, possess a battery of enzymes to exploit this resource, releasing monosaccharides from mucins (Tailford et al., 2015).

### Mucin Glycan Metabolism in Lactic Acid Bacteria

*In vitro* growth experiments using mucins as a carbon source have demonstrated a very limited capacity to use the carbohydrate moiety of these proteins by lactobacilli (Sanchez et al., 2010), although growth in the presence of mucin induced proteomic changes in the bacteria (Celebioglu et al., 2017). Furthermore, intestinal lactobacilli are characterized by the presence of multiple attachment proteins that interact with mucins [e.g., adhesins containing Mub domains (Etzold et al., 2014)] or even carry specialized pili for mucin binding (von Ossowski et al., 2010). Some bifidobacterial species able to use a wide variety of HMOs (e.g., *B. longum* subsp. *infantis*) grow poorly on mucins, possibly because most of their glycosidases are intracellular, thus limiting their capacity to attack mucins. Other bifidobacteria less specialized in HMOs exploitation are better equipped to attack *O*-glycosylated proteins and they can grow on mucins from different origins as a carbon source. The same bifidobacterial extracellular enzymes able to trim terminal L-fucose and sialic acid residues in HMOs (α-L-fucosidases and sialidases) are responsible for the release of these molecules from mucins. A previously identified membrane-bound β-*N*-acetylglucosaminidase from *B. bifidum*, BbhII, has been recently shown to possess sulfoglycosidase activity, being able to release GlcNAc 6-sulfate, which is commonly found in *O*-linked glycans from mucins (Katoh et al., 2017). *B. bifidum* is not able to use this sulfated carbohydrate but its release may contribute to the coordinated degradation of glycans by the microbial community and participate in the cross-feeding at the intestinal mucosa. Notwithstanding, and contrarily to specialized HMOs consumers (i.e., *B. longum* subsp. *infantis*), *B. bifidum* strains are specially adapted to the use of *O*-glycans from mucins and, similar to *Bacteroides* (Marcobal et al., 2011), they exploit this capacity for the use of HMOs. Accordingly, *B. bifidum* JCM 1254 and *B. bifidum* JCM 7004 possess α-*N*-acetylglucosaminidase activity (Shimada et al., 2015). This specialized enzyme cleaves GlcNAcα1-4Gal bonds present at non-reducing termini that are typically found in gastroduodenal mucins, being their cleavage essential as a first step in the utilization of glycans from mucins. The corresponding enzyme, AgnB, has been characterized from *B. bifidum* JCM 1254. It is an extracellular and membrane-anchored glycosidase, with a GH89 catalytic domain and four carbohydrate binding modules of the CBM32 type. These domains participate in binding the enzyme to GlcNAcα1-4Gal epitopes in *O*-glycans, enhancing its activity toward multivalent substrate such as mucins (Shimada et al., 2015). Uncharacterized GH89 enzymes are found in other intestinal bacteria such as *Bacteroides* spp. and *A. muciniphila*, for which they could play a relevant role in obtaining carbohydrates from host glycans (Shimada et al., 2015). In addition to this, other specific glycosidases are involved in carbohydrate release from mucins in bifidobacteria. In *B. longum* JCM 1217 the extracellular endo-α-*N*-acetylgalactosaminidase EngBF (GH101) releases GNB from core-1 structures predominant in gastroduodenal mucins by hydrolyzing the GalNAcα1Ser/Thr bond (Fujita et al., 2005). This disaccharide can be further metabolized via its transport through a dedicated ABC transporter and hydrolysis by the intracellular 1,3-β-galactosyl-*N*-acetylhexosamine phosphorylase present in LNB-GNB metabolic clusters. In *B. bifidum* JCM 1254 two different pathways for assimilating carbohydrates from *O*-glycosylated proteins coexist (Kiyohara et al., 2012). Core-1 type *O*-glycans are hydrolyzed by and EngBF enzyme. By the contrary, core-3 intestinal mucins can be the target for a concerted action of extracellular sialidases (SiaBb), β-galactosidases (BbgIII) and β-*N*-acetylhexosaminidases (BbhI and BbhII) that also participate in the hydrolysis of HMOs. This renders GalNAcα1-Ser/Thr, also called Tn-antigen. This molecule is taken up by an as yet non-identified transporter and cleaved by the intracellular α-*N*-acetylgalactosaminidase NagBb that hydrolyzes the glycosidic bond between GalNAc and Ser/Thr amino acids (Kiyohara et al., 2012). NagBb constitutes the first characterized member of the new GH129 family and the elucidation of its structure revealed the differences with GH101 enzymes regarding mechanism and substrate binding (Sato et al., 2017).

Genome inspection in *B. longum* subsp. *longum*, *B. longum* subsp. *infantis* and *B. breve* reveals that the presence of genes encoding endo-β-*N*-acetylglucosaminidases (from GH18 and GH85 families) correlates with the capacity to use *N*-glycans from glycoproteins (Garrido et al., 2012a). The enzyme EndoBI-1 (GH18) from *B. infantis* ATCC 15697, EndoBI-2 (GH18) from *B. infantis* SC142 and EndoBB (GH85) from *B. longum* DJO10A have been characterized (Garrido et al., 2012a). They contain one or two transmembrane helices and they are able to deglycosylate highly glycosylated bovine RNaseB. EndoBI-1 and EndoBI-2 also deglycosylate human and bovine lactoferrin. EndoBI-1, a constitutively expressed and heat-stable (95°C, 5 min) enzyme, was shown to cleave the glycosidic bond of ChbNAc in proteins containing high mannose or other complex *N*-glycans (Garrido et al., 2012a; Karav et al., 2015). In lactobacilli no endo-β-*N*-acetylglucosaminidases releasing glycans from *N*-glycosylated proteins have been characterized. However, in *L. sakei* 23K the *asnA* gene codes for a putative glycosyl asparaginase that would cleave GlcNAc-asparagine bonds in *N*-glycoproteins. This gene is induced after growth in meat and its mutation causes a reduced performance during raw-sausage fermentation (Hufner et al., 2007), suggesting that it is implicated in scavenging carbohydrates from proteins in this fermentation niche. A homolog to *asnA* is present in the *L. casei* BL23 genome (LCABL_29290, (Maze et al., 2010) showing 67% identity to the *L. sakei* counterpart at the protein level. The participation of these genes in the release of carbohydrate moieties from *N*-glycosylated proteins still needs to be proven but, as occurred with other complex oligosaccharides, lactobacilli probably need the coordinated action of different glycosidases provided by other commensal microorganisms to exploit these resources. These would render simplified substrates from *N*-glycans that are adequate for their transport and hydrolytic machinery. Similarly, the *gnb* operon of *L. casei* would be designed for the utilization of core-1 *O*-glycans (Bidart et al., 2014), although they must be previously processed by other members of the microbiota in order to render GNB.

### Structural Characteristics of Glycolipids

**FIGURE 4:**
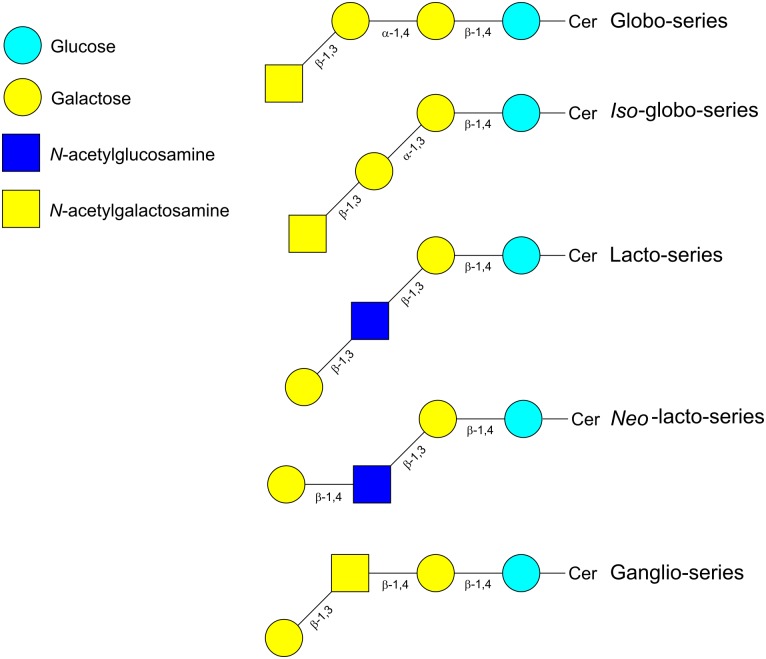
Schematic representation of the different types of glycosphingolipids. Cer, ceramide.

**FIGURE 5:**
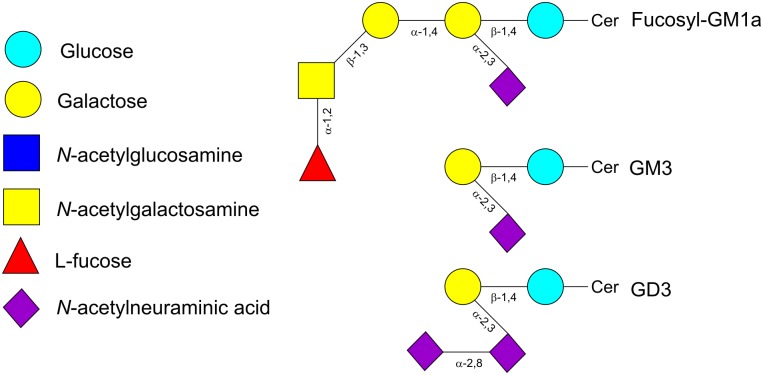
Schematic representation of the gangliosides fucosyl-GM1a, GM3 and GD3. Cer, ceramide.

Glycolipids are structurally diverse glycoconjugates present in organisms across all three domains of life. The main glycolipid species found in plants and bacteria are glycoglycerolipids, while in vertebrates and insects are glycosphingolipids (GSLs) (Kopitz, 2017). Glycoglycerolipids usually contain one or two sugars linked to diacylglycerol whereas the lipid backbone of GSLs is ceramide (Cer). Ceramides consist of a molecule of sphingosine linked via amide linkage to a fatty acid molecule. The GSLs have either glucose (GlcCer) or galactose (GalCer) attached to the hydrophobic ceramide moiety by a β-linkage with the terminal hydroxyl group of sphingosine. Addition of Gal to GlcCer produces lactosylceramide (LacCer), whose elongation with further sugars results in the different types of GSLs (**Figure 4**). Thus, the addition of Gal, GlcNAc or GalNAc with specific α- or β-bonds leads to the globo-, *iso*-globo-, lacto-, *neo*-lacto- or ganglio-series (Merrill, 2011). Further subdivision of gangliosides (G) is based on the addition of Neu5Ac; thus depending on the number of the Neu5Ac residues there are several subfamilies (GA = 0, GM = 1, GD = 2, GT = 3, GQ = 4, GP = 5, GH = 6, GS = 7). The two-letter nomenclature is followed by a number (n) that allows to calculate the length of the neutral chain with the formula 5-n. Additionally, depending on the position of the Neu5Ac residue they are divided in a-, b-, or c- series (Ryan et al., 2013; Kopitz, 2017). Fucose can also be added to produce fucosyl-gangliosides (**Figure 5**) (Merrill, 2011). As with other glycoconjugates, the glycan moiety structure of glycolipids depends upon the expression of histo-blood group glycosyltransferases from the Secretor, Lewis and ABO systems (Angstrom et al., 2004).

Glycosphingolipids constitute a significant proportion of the total lipids of the GI tract epithelial cells (Jennemann and Grone, 2013). They are involved in many physiological functions, including cell-cell communication, cell differentiation, endocytosis and vesicular transport (Jennemann and Grone, 2013). Their glycan moieties can interact with proteins like enzymes, antibodies and lectins (protein–glycan recognition) or with related glycans (glycan–glycan recognition). They can also be used as receptors by bacteria, bacterial toxins and viruses (Cho and Blaser, 2012) and altered GSLs structures can be associated with immunological diseases and cancer (Furukawa et al., 2006).

The glycolipid fraction present in human milk is constituted mainly by gangliosides and they are usually associated with membranous structures of milk, especially with the milk fat globule membrane (MFGM) (Newburg, 1996). The concentration of gangliosides in human colostrum and mature milk is similar and around 9 mg/l (Pan and Izumi, 2000) but, the composition changes through lactation. The most abundant ganglioside on human colostrum is disialoganglioside GD3, while in mature milk is monosialoganglioside GM3 (**Figure 5**). Other gangliosides such as GM1 and GM2, and neutral glycolipids as GlcCer, GalCer, LacCer, globotriaosylceramide (Gb3), and globotetraosylceramide (Gb4) (**Table 2**) are also present in mature milk at lower concentrations than GM3 (Newburg, 1996). Milk gangliosides are known to compete with pathogen binding receptors thus preventing the attachment of enteropathogens such as enterotoxigenic *E. coli*, *Campylobacter jejuni*, *Listeria monocytogenes*, *Salmonella enterica*, *Shigella sonnei*, and *Helicobacter pylori* (Newburg, 2013; Peterson et al., 2013). As well, toxins produced by pathogenic bacteria are neutralized by specific gangliosides; GM1 bound to *E. coli* and *C. jejuni* heat labile enterotoxins and cholera toxin, and Gb3 to Shiga toxins from *S. dysenteriae* and enterohemorrhagic *E. coli* (Newburg, 2013; Peterson et al., 2013). Likewise, glycolipids from human milk showed antiviral activity against human respiratory syncytial virus, enterovirus 71 and HIV (Peterson et al., 2013). In addition to these activities, a number of studies have also revealed that specific bifidobacteria are able to catabolize gangliosides from milk, suggesting that these glycolipids may play a significant role as prebiotics in the infant gut (Rueda et al., 1998; Lee et al., 2014).

### Glycolipid Metabolism by *Bifidobacterium*: Glycosphingolipids (GSLs)

Glycosidases isolated from gut bacteria and involved in the degradation of the sugar chains of GSLs were described first in *Ruminococcus* and *Bifidobacterium* genera (Larson et al., 1988; Falk et al., 1990). These enzymes are extracellular α- and β-glycosidases with activity on the lacto-, *neo-*lacto- and ganglio-series of GSLs. The analysis of the degradation patterns of different GSLs, together with the production of LacCer as the major end product, by enzymes mixtures from *B. infantis* and *B. bifidum* species, revealed the action of α-1,2/α-1,3-L-fucosidases, α-2,3/α-2,8-*N*-acetylneuraminidases (also called α-2,3/α-2,8-sialidases), β-1,4-galactosidases and endo-β-1,3-*N*-acetylglucosaminidases (Falk et al., 1990). Recently, it has been described that the α-1,2-L-fucosidase AfcA isolated from *B. bifidum* is able to release fucose from fucosyl-GM1 ganglioside (Sugiyama et al., 2017). As previously indicated, lacto-*N*-biosidases LnbX and LnbB showed activity toward the glycan parts of globosides and gangliosides. LnbX released GNB from Gb5 and 2′-fucosyl GNB from Globo H oligosaccharides, and LnbB released GNB from Gb5 and GA1 oligosaccharides (Gotoh et al., 2015).

*Bifidobacterium breve*, *B. longum* subsp. *Infantis*, and *B. bifidum* species were able to grow *in vitro* in the presence of bovine milk gangliosides as the carbon source. *B. infantis* and *B. bifidum* utilized GD3 (63 and 100% consumed, respectively) and GM3 (42 and 99% consumed, respectively), whereas *B. breve* did not utilize these gangliosides (Lee et al., 2014). *B. longum* subsp. *longum*, *B. animalis* subsp. *Lactis*, and *B. adolescentis* did not grow in the presence of bovine milk gangliosides, but they hydrolyzed moderate amounts of GD3 (30, 48, and 28% consumed, respectively). Among the last three species, only *B. animalis* subsp. *lactis* consumed a low amount of GM3 (10%). Degradation of gangliosides was not affected by the ceramide chain lengths, suggesting that bifidobacteria specifically recognized the ganglioside glycan moieties rather than their ceramide parts (Lee et al., 2014). Growth of *Bifidobacterium* species with specific gangliosides results in the accumulation in the culture media of distinct LacCer molecules, indicating that the degradation occurs at the outer residues of their glycan group (Lee et al., 2014). *B. infantis* and *B. bifidum* have α-2,8-sialidase activity that released Neu5Ac from GD3 (Lee et al., 2014). As well, the extracellular exo-α-sialidase SiaBb2 from *B. bifidum* hydrolyzed very efficiently the α-2,3 linkage present in different substrates, including gangliosides (Kiyohara et al., 2011), and the intracellular exo-α-sialidases, NanH1 and NanH2, from *B. infantis* showed specificity toward α-2,3 and α-2,6 linkages (Sela et al., 2011). The important role of human milk gangliosides in the establishment of gut bifidobacteria is also supported by clinical studies; these showed that the addition of gangliosides to infant formula resulted in an increment of fecal bifidobacterial counts (Rueda et al., 1998).

### Structural Characteristics of Glycosaminoglycans (GAGs) and Their Impact on Lab Growth

**FIGURE 6:**
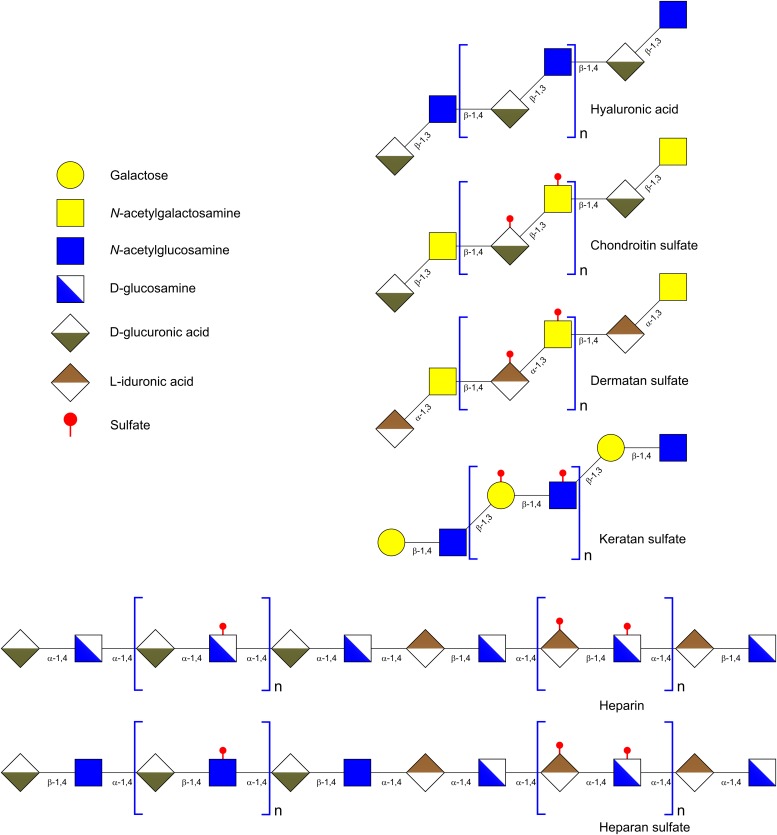
Schematic representation of glycosaminoglycans.

Glycosaminoglycans are linear acidic heteropolysaccharides constituted by repeating disaccharide units, which can be highly sulfated (**Figure 6**) (Zhang et al., 2009). Regarding their monosaccharide composition and structure, GAGs are usually divided into four classes: (1) non-sulfated hyaluronic acid (HA); (2) chondroitin sulfate (CS) and dermatan sulfate (DS); (3) heparin (Hep) and heparan sulfate (HS); and (4) non-uronic acid polymer keratan sulfate (KS) (Jackson et al., 1991). HA is composed of a disaccharide consisting of D-glucoronic acid (GlcA) and GlcNAc. The disaccharide units are linked through β-1,4 glycosidic bonds (**Table 2**). HA occurs as a free polysaccharide, unlike the rest of GAGs which are covalently attached to a protein core forming proteoglycans (Jackson et al., 1991). CS and DS contain mainly repeating units of GalNAc and an uronic acid, GlcA in CS and L-iduronic acid (IdoA) in DS (**Table 2**). The disaccharide moiety may be un-sulfated, mono- or di-sulfated. GlcNAc can be sulfated at positions 4 and 6 and GlcA at position 2. These units are linked to each other through a β-1,4 glycosidic bond. Within GAGs, Hep and HS possess the most complex structures. The hexosamine of the disaccharide unit can be D-glucosamine (GlcN) or GlcNAc and the uronic acid can be GlcA or IdoA. The monosaccharides of the repeating unit are linked through β-1,4 or α-1,4 linkages. These units are linked to each other through an α-1,4 glycosidic bond (**Table 2**) (Sampaio et al., 2006). Finally, KS contained galactose instead of uronic acid and the disaccharide units are linked through β-1,3 bonds (**Table 2**) (Coppa et al., 2011).

Glycosaminoglycans are present at mammalian cell surfaces and in the extracellular matrix, where they play important roles in many physiological processes such as regulation of cell proliferation, cell differentiation and in innate defense responses, among others (Hill et al., 2013). Several studies also described that GAGs participated in pathogen adhesion and infection (Aquino et al., 2010). Additionally, GAGs are available in the gut as nutrients for the microbiota. It has been recently reported that the human gut symbiont *Bacteroides thetaiotaomicron* possessed an array of enzymes, with complementary functions, that allowed this bacterium to degrade the highly variable sulfated structures of Hep and HS (Cartmell et al., 2017). The degradation of these complex carbohydrates is carried out by a collection of polysaccharide lyases belonging to PL12, PL13, and PL15 families of the CAZy database, a glycosidase of the GH88 family and several sulfatases (Cartmell et al., 2017). The ability of lactobacilli and bifidobacteria to utilize GAGs as carbon sources remains to be determined. Several studies have recently demonstrated that orally administrated Hep, heparosan (precursor of Hep and HS) (Duan et al., 2013), CS-derived oligosaccharides (Shang et al., 2016b) and KS (Shang et al., 2016a) have potential prebiotic properties, as they were able to modulate the gut microbiota by stimulating the growth of *Lactobacillus* species. However, an *in vitro* fermentation model with fucosylated CS and human fecal microbiota resulted in a reduction of *Enterobacteriaceae* and *Lactobacillus* cells, while the amounts of *Clostridium*, *Bacteroides, Prevotella*, and *Bifidobacterium* cells increased (Wei et al., 2017). Therefore, further studies are needed to understand the role of lactobacilli and bifidobacteria on the metabolism of GAGs in the human gut.

Human milk contains GAGs mainly constituted by CS (about 55%), Hep (about 40%), whereas DS and HA made up the remaining 5% (Coppa et al., 2011). The concentration of HA ranges from about 100–1,000 ng/ml, the highest values being reached during the first 2 months after delivery (Hill et al., 2013). Similarly to HMOs, milk GAGs have been shown to prevent the binding of the enteropathogenic *E. coli* serotype O119 and *Salmonella fyris* to the intestinal mucosa (Coppa et al., 2016). As well, a CS milk fraction was able to avoid the binding of the HIV envelope glycoprotein gp120 to the cellular CD4 receptor (Newburg et al., 1995). HA isolated from human milk enhanced the expression of the antimicrobial peptide β-defensin 2 in cultured colonic epithelial cells and protected them against *S. typhimurium* infection. These results together with *in vivo* experiments demonstrated that host responses to HA are dependent on TLR4 and CD44 signaling receptors. Therefore, HA in human milk might have an important effect on innate immune defense at the GI tract of newborns. Human milk GAGs are not digested and reach the newborns colon (Maccari et al., 2016); however, analysis of GAGs in the feces of infants showed only residual amounts compared with the GAGs content in the ingested milk, indicating a high bioavailability (Maccari et al., 2016). As described above, *in vivo* and *in vitro* models used to analyze the effect of dietary GAGs on the modulation of gut microbiota suggested that members of the *Lactobacillus* and *Bifidobacterium* genus may be able to metabolize these glycans. Since species of these genus are members of the infant GI microbiota (Martin et al., 2007; Albesharat et al., 2011; Turroni et al., 2012), the milk GAGs might function as metabolic substrates to feed lactobacilli and bifidobacteria species present at the infants gut.

Although the above studies shed light on the potential roles of GAGs to modulate the gut microbiota, additional investigations are still needed to fully understand the biological functions and metabolic fate of host-derived GAGs in the human GI tract.

## Conclusion

Bifidobacteria and lactobacilli are widely used by the food industry as probiotics or health-promoting bacteria in functional foods. Therefore, an understanding of the mechanisms utilized by these microorganisms to survive in the gut will help to understand their exact role that they play in health and disease development. Colonization of the gut by probiotic or commensal members of these genera relies on their saccharolytic capacity as revealed by the remarkable carbohydrate gene repertoires found in their genomes. However, the exact functions of most putative enzymes encoded by these genes remain to be elucidated. Similarly, carbohydrate transport systems and regulatory mechanisms, especially in bifidobacteria, are still poorly characterized. Three different strategies of host-glycan utilization have been described for strains belonging to the *Bifidobacterium* genus: (i) internalization of intact oligosaccharides by ABC transporters, and subsequent degradation by intracellular glycosidases; (ii) secretion of glycosyl hydrolases and internalization of the resulting monosaccharide or disaccharide residues and (iii) scavenging, whereby some *Bifidobacterium* strains metabolize simple sugars released from glyco-complexes by other members of the gut microbiota. Regarding the genus *Lactobacillus*, knowledge about host-glycans metabolism is mostly restricted to strains belonging to the *L. casei*–*paracasei*–*rhamnosus* phylogenetic group. Members of this group represent the only lactobacilli where catabolic routes for mucosa and HMOs oligosaccharides have been found. The simple structure of these oligosaccharides, mainly disaccharides, and the description of only one extracellular enzyme involved in HMOs degradation, suggest that lactobacilli have a limited capacity to directly access mucin or complex HMOs as carbon sources. Therefore, lactobacilli must rely on the release of oligosaccharides from glyco-complexes by primary saccharolytic bacteria through the activity of extracellular glycosyl hidrolases. Although a number of studies have addressed the ecological implications of glycan utilization in the intestinal microbiota, knowledge on the metabolic interactions among members of the intestinal microbiota is still limited, and the relevance that they would have in the establishment of symbiotic relationships with other members of the gut microbiota and the host deserve further attention.

## Author Contributions

MZ, VM, and MY contributed to the writing, critical revision of the manuscript, and approved its final version.

## Conflict of Interest Statement

The authors declare that the research was conducted in the absence of any commercial or financial relationships that could be construed as a potential conflict of interest.

## Footnotes

1 http://mbgd.genome.ad.jp/
